# Effects of Time of Day and Sleep Deprivation on Motorcycle-Driving Performance

**DOI:** 10.1371/journal.pone.0039735

**Published:** 2012-06-28

**Authors:** Clément Bougard, Stéphane Espié, Bruno Larnaudie, Sébastien Moussay, Damien Davenne

**Affiliations:** 1 INSERM ERI27, Université de Caen, Caen, France; 2 IFSTTAR IM, Paris, France; 3 IRBA Département Environnements Opérationnels, Brétigny-sur-Orge, France; 4 IEF AXIS, Université Paris-Sud, CNRS UMR 8622, Orsay, France; Vanderbilt University, United States of America

## Abstract

The aim of this study was to investigate whether motorcycle handling capabilities – measured by means of the efficiency of emergency manoeuvres – were dependent on prior sleep deprivation and time of day. Twelve male participants voluntarily took part in four test sessions, starting at 6 a.m., 10 a.m., 2 p.m., and 6 p.m., following a night either with or without sleep. Each test session comprised temperature and sleepiness measurements, before three different types of motorcycling tests were initiated: (1) stability in straight ahead riding at low speed (in “slow motion” mode and in “brakes and clutch” mode), (2) emergency braking and (3) crash avoidance tasks performed at 20 kph and 40 kph. The results indicate that motorcycle control at low speed depends on time of day, with an improvement in performance throughout the day. Emergency braking performance is affected at both speeds by time of day, with poorer performance (longer total stopping distance, reaction time and braking distance) in the morning, and also by sleep deprivation, from measurements obtained at 40 kph (incorrect initial speed). Except for a tendency observed after the sleepless night to deviate from the initial speed, it seems that crash avoidance capabilities are quite unaffected by the two disturbance factors. Consequently, some motorcycle handling capabilities (stability at low speed and emergency braking) change in the same way as the diurnal fluctuation observed in body temperature and sleepiness, whereas for others (crash avoidance) the participants were able to maintain their initial performance level despite the high levels of sleepiness recorded after a sleepless night. Motorcycle riders have to be aware that their handling capabilities are limited in the early morning and/or after sleep deprivation. Both these situations can increase the risk of falls and of being involved in a road accident.

## Introduction

The analysis of powered two-wheeler (PTW) handling capabilities is of major interest for road safety. PTWs are a popular form of transport, representing between 3 and 15% (depending on the country) of all the vehicles in circulation in Europe [1]. Moreover, it is well known that PTW riders are more at risk of being killed in an accident than other road users [2]. For example, in France the risk of fatal injury in an accident is about 21 times higher for a PTW rider than for a motorist [3]. A road traffic accident in which a PTW rider is involved is generally caused by the interaction between various factors [4]. Among these can be listed the dynamic properties of the PTW [5], PTW riders being inconspicuous [6], direct exposure to environmental conditions, e.g., meteorological conditions and poor road surfaces [7], and handling difficulties such as avoidance or emergency braking [8]–[9].

Epidemiological data suggests that fatal accidents and dangerous behaviour patterns (such as alcohol and/or drug consumption, riding without a headlight, and not wearing a helmet) mainly occur at weekends and at night [10]–[18]. Accordingly, even if traffic density is more important during the day, PTW riders are more at risk of being injured or killed in an accident at night time, regardless of the country under consideration (Brazil, France, India, Italy, and Thailand have all been studied) [10], [15], [19]–[24]. More precisely, the risk of injury seems to increase by a factor of 1.5 from day to night [16]. As previously suggested [25], in addition to the factors mentioned above, a reduced level of vigilance during the night may contribute to the rider showing less anticipatory behaviour and reactivity. It has been shown that the most serious accidents are brought on by mistakes in attention or excessive sleepiness, which reach a high level after midnight, and result in a lack of reaction by the PTW rider before a crash [26]–[28]. Consequently, the decrease in the level of vigilance is correlated with the gravity of the accident [14].

When emergency manoeuvres are chosen, braking and/or avoidance manoeuvres are the most frequently used [3], [14], [27], [29]. However, these manoeuvres are particularly complex and are less efficient in PTW riding than in car driving [30]–[31]. Moreover, these manoeuvres are not correctly carried out in more than half of the cases studied. On most occasions when the manoeuvre is chosen it is not even appropriate for the constraints of the situation [27]. Due to the dynamic characteristics of PTWs, many physical, cognitive and psychomotor resources (such as balance, visual perception, reaction time and motor coordination) are required in order to carry out these manoeuvres adequately [32]–[34]. Many studies have shown that these different resources develop concomitantly with the level of vigilance, and thus vary with time of day and sleep deprivation [35]–[37].

At present, the evaluation of the respective and/or combined effects of time of day and total sleep deprivation (TSD) on PTW riding has only been poorly studied [38]–[39]. However, in regards to (i) automobile driving studies and (ii) the temporal distribution of PTW accidents, it seems that PTW riding performance evolves concomitantly with body temperature and/or sleepiness rhythm. The aim of our study was to investigate whether PTW handling capabilities – measured by means of the efficiency of emergency manoeuvres – are dependent on prior sleep deprivation and/or time of day.

## Materials and Methods

### Ethics Statement

The study protocol complied with the tenets of the Declaration of Helsinki and was approved by the local ethics committee (Comité de Protection des Personnes Nord-Ouest III, France, n° 2007-A00581-52).

### Participants

Twelve male participants (age: 22.0±2.3 years; height: 179.2±8.9 cm; weight: 80.4±21.9 kg) voluntarily took part in the experiment and signed an informed consent before being included in this study. To guarantee the homogeneity of the sample, particular attention was paid to motorcycling experience (participants had held a driving licence for 4.1±1.8 years) and to the participant’s chronotype, according to their answers to the Horne and Östberg questionnaire [40]. All the participants were “intermediate type”.

### Study Design

**Figure 1:**
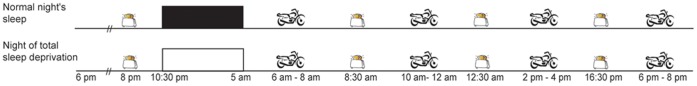
Experimental protocol. Each participant participated in two days of test sessions, organised after either a normal night’s sleep or a night of TSD. During these days, standardised meals were provided between test sessions.

As illustrated in Figure 1, each participant was evaluated during four test sessions, set up at 6 a.m., 10 a.m., 2 p.m., and 6 p.m., following a night with or without sleep, in a random order. The two days of testing were separated by a period of 1 week to allow for recovery from the night of TSD. For each of the two sleep conditions, the tests described in the following section were carried out once by each of the 12 participants at each time of day.

Each participant arrived at the laboratory (which had room temperature of 21.6±0.8°C) at 7 p.m. the day before the test, and ate a standardised meal. When the participants were evaluated following a night of normal sleep, they were asked to go to bed at 10∶30 p.m. in order to guarantee a minimum of 6 hours in bed. They were woken by an experimenter at 5 a.m. Under these conditions, sleep duration conformed closely to the participants’ usual sleeping habits, since participants reported during the inclusion visit that their mean sleep duration was 7 hours. For the night of TSD, the participants remained in the presence of an experimenter and were not allowed to lie down. During this night of TSD, the participants were only allowed to take part in activities not involving any physical load or excitement (such as reading, watching a movie, and playing cards), and energising and stimulant drinks (coffee, tea, etc) were not allowed [41].

A standardised breakfast was provided at 8∶30 a.m., just after the experimental session at 6 a.m. [42], in order to limit inter-individual variability of the results [43]. A standardised meal was provided for lunch at 12∶30 p.m.

### PTW Riding Sessions

All the PTW riding tests were carried out under covered parking with controlled environmental conditions (light and temperature). The sessions took place on a lane 100 m long and 3 m wide divided into two zones of 40 m (a preparation zone and an exercise zone). At the beginning and the end of the lane, a 10 m zone was provided to make a U-turn.

**Figure 2:**
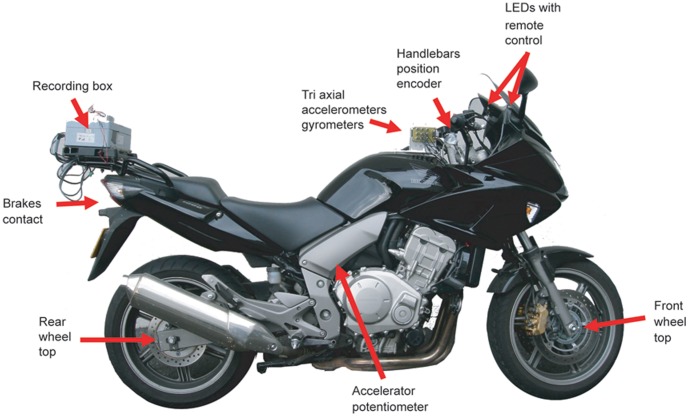
Location of the different measurement devices.

The same instrumented motorcycle (1000 CBF, Honda®) was used for all the tests. This PTW was equipped with ABS (anti-braking system)/CBS (combined braking system). As depicted in Figure 2, various transducers already used in a previous study [44] were mounted on the PTW to record the actions of the PTW rider. The positions of these transducers, and the measurements they recorded, were as follows: (i) the handlebars (direction), (ii) the throttle (acceleration/deceleration), (iii) the brake levers, (iv) the front and rear wheel position (distance covered, speed), and (v) dynamics information (yaw, roll, pitch). All the data were recorded at 1000 Hz frequency and to an accuracy of 4 µs. To limit the weight and inertial effects of the system, the recording box was fixed directly at the rear of the PTW.

For the experiments, a white LED was added on each side of the front panel of the PTW, positioned at the periphery of the field of view. The lights could be activated at any time by the test coordinator via a remote control. Therefore the trigger signal for starting a manoeuvre was quite “unexpected” for the riders.

### Outcome Measures

During the first 15 minutes of each test session, participants stayed in a supine position. Their oral temperature was measured at the end of this period by an experimenter using a digital clinical thermometer (Omron®, accuracy: 0.05°C) inserted sublingually for at least 3 minutes. The participants then completed the Epworth Sleepiness Scale [45] in a quiet room isolated from external stimuli, before being included in the motorcycling tests.

Participants self-estimated their propensity to sleepiness in the context of eight circumstances of daily life (e.g. watching television or while seated), using the Epworth Sleepiness Scale. The degree of sleepiness was evaluated for each circumstance on a discontinuous scale, graded from 0 (non-existent risk) to 3 (significant risk). The sum of the eight scores was used as an indicator of the diurnal level of sleepiness.

PTW riding performance was evaluated using three different types of tests presented in a random order. To avoid any performance improvement during the experiment due to a practice effect [46], all the participants were trained in both laboratory and field tests during a pre-experimental session set up a week before at 1 p.m., to obtain stabilisation of their performances.

**Figure 3:**
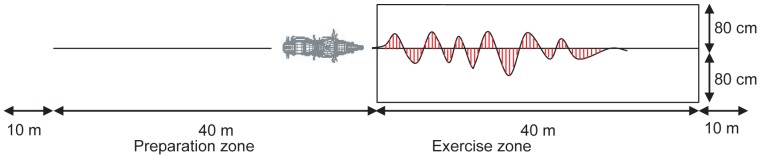
Test set-up to evaluate stability using deviation calculations when driving straight ahead at low speed.

The first test aimed to evaluate stability in straight ahead riding at low speed. Participants had to ride on a line of 8 cm in width painted on the ground, using only first gear and without touching the accelerator or the brakes, in the “slow motion” mode. In the “brakes and clutch” mode, the participants could use those instruments in order to ride as slowly as possible while keeping the track of the PTW on the line. For the “slow motion” mode, as the speed was fixed by the engine, the performance was evaluated by calculating the sum of the deviations of the PTW track from the line (Figure 3) by means of the values of the handlebar position transducer (measured in °). In addition, the average speed was calculated for the “brakes and clutch” mode.

**Figure 4:**
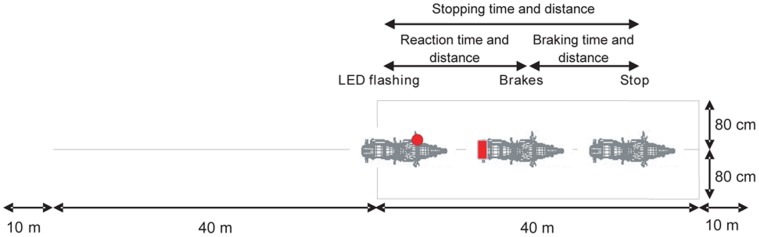
Emergency braking test and recorded measurements.

The second test consisted of emergency braking and was carried out at either 20 kph (12.43 mph) or 40 kph (24.86 mph) with the brake lever and pedal in a “ready-to-brake” position (foot just above the rear wheel brake pedal and fingers beyond the front wheel brake lever). Participants had to achieve the required speed before entering the exercise zone. From the entrance to the exercise zone, one of the two LEDs was switched on in a random way by the experimenter (by means of the remote control). At this signal, the participant had to brake in order to stop the PTW as soon as possible (Figure 4). The stopping time and distance were retained as performance indices. The combination of the different encoders also allowed for the calculation of reaction time and distance, and of braking time and distance. Moreover, the average braking deceleration was calculated using the following formula: average braking deceleration  =  initial velocity^2^/(2×braking distance).

**Figure 5:**
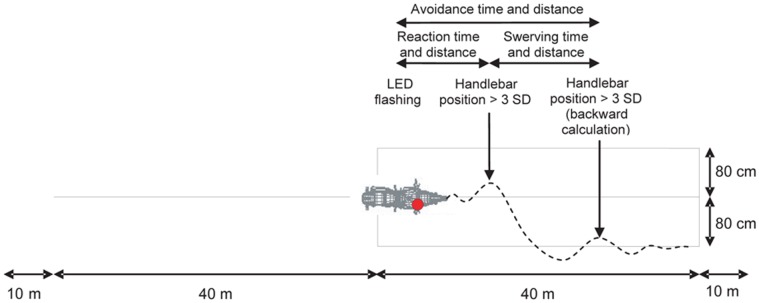
Crash avoidance test and recorded measurements.

The third test consisted of a crash avoidance manoeuvre. As for the braking test, the expected speed had to be attained before entering the exercise zone. At the beginning of that zone, participants had to adjust the track of the PTW to the median line (as for the stability test). Then one of the two LEDs was switched on by the experimenter (by means of the remote control) and the participant had to adjust the track of the PTW to the line on the same side as the LED, as soon as possible. To increase the difficulty, the participants also had to adjust the track of the PTW to the line on the opposite side to that of the LED which had been switched on. The order in which participants had to perform the manoeuvre on the side indicated and on the opposite one, and also which LED was switched on (the left one or the right one) were randomly chosen. The total time and distance for the avoidance manoeuvre were retained as performance indices (Figure 5). The combination of the different encoders permitted the calculation of reaction time and distance, and also swerving time and distance. In order to do so, the mean and the standard deviation of the handlebar position were calculated for the exercise zone. First, the reaction time and the matching distance corresponded to a handlebar position greater than 3 standard deviations (SD) (beginning of the turn) after the LED was switched on. Second, the file was read backward from the stop of the motorcycle at the end of the exercise zone, and the point at which the handlebar position was greater than 3 SD (end of the adjustment of the trajectory) allowed an estimate of the swerving time and distance to be made.

### Data Processing and Analysis

The data recorded in laboratory tests during the eight test sessions were analysed by a 2 (sleep condition: normal night; sleep deprivation) × 4 (time of day: 6 a.m.; 10 a.m.; 2 p.m. and 6 p.m.) repeated-measure analysis of variance (ANOVA). For the motorcycling tests, stability at low speed and emergency braking were evaluated at two different speeds. Consequently, these data were analysed by a 2 (speed: for the stability on low speed test (slow motion; brakes and clutch) and for the emergency braking test (20 kph; 40 kph)) × 2 (sleep condition: normal night; sleep deprivation) × 4 (time of day: 6 a.m.; 10 a.m.; 2 p.m. and 6 p.m.) repeated-measure analysis of variance (ANOVA). For the crash avoidance test, the data were analysed by a 2 (instruction given: same side; opposite side) × 2 (speed: 20 kph; 40 kph) × 2 (side on which the LED was switched on: right; left) × 2 (sleep condition: normal night; sleep deprivation) × 4 (time of day: 6 a.m.; 10 a.m.; 2 p.m. and 6 p.m.) repeated-measure analysis of variance (ANOVA).

For all the collected data, the condition of sphericity was tested (Mauchly’s test). The *p*-value levels were corrected for possible deviations from sphericity by means of the Huynh–Feldt epsilon (*ε*). We report the uncorrected degrees of freedom, the ε value and the *p*-value according to the corrected degrees of freedom. When significant differences were observed, a post hoc analysis was then performed with a Fischer–Snedecor least significant difference (LSD) test.

In addition, in order to test our a priori hypothesis which postulates that diurnal fluctuations, i.e. the difference between the minimal and maximal values recorded for temperature and sleepiness (in this case, between the values recorded at 6 a.m. and 6 p.m.), are determined by the previous sleep condition (normal or sleep deprivation), planned comparisons were applied. Similarly, in order to check the a priori hypothesis, according to which the temporal evolution of the variable studied across the day was modified by the lack of sleep, planned comparisons were applied to the values obtained at each test session organised at the same time of day, according to the previous sleep condition.

All differences were considered as significant for *p*-value <0.05. For each significant effect, we estimated the size effect using the partial eta squared (partial *η^2^*).

## Results

### Temperature

**Figure 6:**
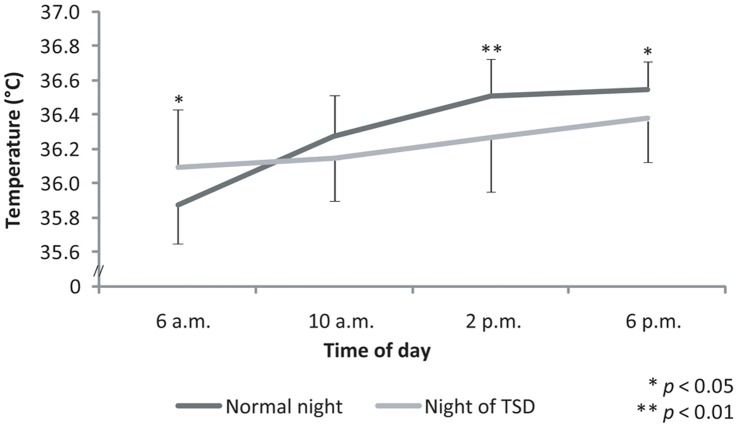
Oral temperature recorded at 6 a.m., 10 a.m., 2 p.m., and 6 p.m. after either a normal night’s sleep (Normal night) or a night of TSD (Night of TSD).

An effect of “time of day” on the level of body temperature was observed (F_(3,33)_ = 18.77; *ε* = 0.85; *p*<0.001; partial *η^2^* = 0.63). The levels of temperature increased throughout the day. Thus, temperature levels recorded at 6 a.m. (35.98±0.30°C) were significantly lower than those recorded at the other times of the day (Figure 6). Temperature levels observed at 10 a.m. (36.21±0.25°C) were significantly lower than those recorded at 2 p.m. (36.39±0.29°C) and at 6 p.m. (36.47±0.23°C).

An interaction effect was observed between “sleep condition” and “time of day” (F_(3,33)_ = 8.63; *ε* = 1.00; *p*<0.001; partial *η^2^* = 0.44). The analysis of the planned comparisons indicated that the amplitude of the diurnal fluctuation observed following a normal night’s sleep decreased with TSD (F_(1,11)_ = 11.43; *p*<0.01), mainly because of an increase in the values recorded in the early morning. The analysis of the planned comparisons reported that the levels of temperature recorded at 6 a.m. (F_(1,11)_ = 7.23; *p*<0.05) following a normal night’s sleep were significantly lower than those recorded at the same time of day after TSD (35.87±0.22°C *vs* 36.09±0.34°C). Temperature levels observed at 2 p.m. (F_(1,11)_ = 16.43; *p*<0.01) and at 6 p.m. (F_(1,11)_ = 4.91; *p*<0.05) following a normal night’s sleep were higher than those found after TSD [(36.51±0.22°C *vs* 36.27±0.31°C), (36.55±0.16°C *vs* 36.38±0.26°C), respectively]. On the other hand, the measurements carried out at 10 a.m. (F_(1,11)_ = 2.51; *p* = 0.14) did not depend on the previous sleep condition (36.27±0.24°C *vs* 36.09±0.34°C).

### Epworth Sleepiness Scale

**Figure 7:**
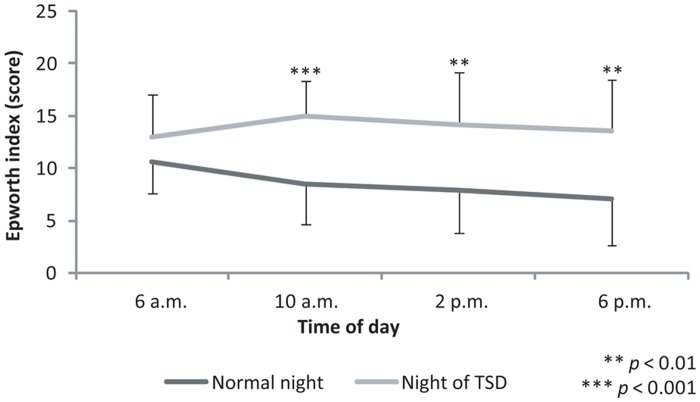
Scores obtained on the Epworth Sleepiness Scale at 6 a.m., 10 a.m., 2 p.m., and 6 p.m. after either a normal night’s sleep (Normal night) or a night of TSD (Night of TSD). The higher the index, the higher the sleepiness level.

The statistical analysis indicated a significant effect of “sleep condition” on the estimated levels of sleepiness (F_(1,11)_ = 27.46; *ε* = 1.00; *p*<0.001; partial *η^2^* = 0.71). Participants estimated that they were less vigilant following TSD (13.92±4.32 points) than after a normal night’s sleep (8.50±3.96 points) (Figure 7).

No interaction effect between “sleep condition” and “time of day” (F_(3,33)_ = 3.11; *ε* = 0.64; *p* = 0.07) was observed. The analysis of the planned comparisons reported that the amplitude of the diurnal fluctuations (6 a.m. *vs* 6 p.m.) of the sleepiness levels was independent of the previous sleep condition (F_(1,11)_ = 3.49; *p* = 0.09). Sleepiness levels at 6 a.m. (F_(1,11)_ = 3.69; *p* = 0.08) were not influenced by the lack of sleep (10.58±3.03 points *vs* 13.00±4.00 points). On the other hand, sleepiness levels evaluated at 10 a.m. (F_(1,11)_ = 22.58; *p*<0.001), 2 p.m. (F_(1,11)_ = 17.49; *p*<0.01) and 6 p.m. (F_(1,11)_ = 17.26; *p*<0.01) following a normal night’s sleep were significantly lower than those recorded at the same time of day after TSD [(8.50±3.89 points *vs* 15.00±3.36 points), (7.83±3.97 points *vs* 14.17±5.04 points), (7.08±4.39 points *vs* 13.50±4.94 points), respectively].

### Motorcycling Performance

#### Stability at low speed

**Figure 8:**
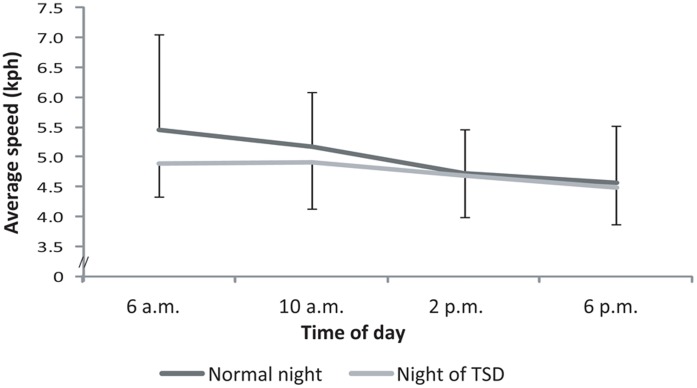
Average speed (kph) of the participants measured in the “brakes and clutch” mode at 6 a.m., 10 a.m., 2 p.m., and 6 p.m. after either a normal night’s sleep (Normal night) or a night of TSD (Night of TSD).

With regards to the average speed, participants rode significantly slower in the “brakes and clutch” mode (4.86±0.93 kph) than in the “slow motion” mode (11.29±0.20 kph) (F_(1,11)_ = 1263.62; *ε* = 1.00; *p*<0.001; partial *η^2^* = 0.99). The statistical analysis indicated a significant effect of “time of day” on the average speed in the “brakes and clutch” mode (F_(3,33)_ = 5.31; *ε* = 0.93; *p*<0.01; partial *η^2^* = 0.33). Recorded speeds were higher at 6 a.m. (5.17±1.20 kph) and at 10 a.m. (5.03±0.85 kph) than at 6 p.m. (4.53±0.79 kph) (Figure 8).

**Figure 9:**
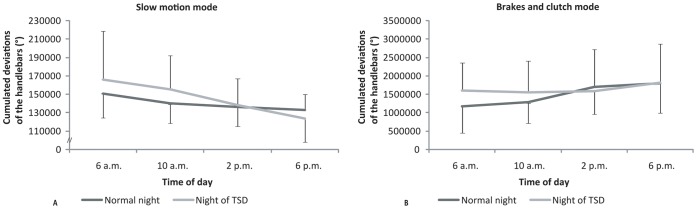
Cumulated deviations of the handlebars (°) in the “slow motion” mode (A) and in the “brakes and clutch” mode (B) at 6 a.m., 10 a.m., 2 p.m., and 6 p.m. after either a normal night’s sleep (Normal night) or a night of TSD (Night of TSD).

Measurements of the cumulated variations depended on an interaction effect between “control mode” and “time of day” (F_(3,33)_ = 7.27; *ε* = 1.00; *p*<0.001; partial *η^2^* = 0.39). In the “slow motion” mode, cumulated deviations were always less important than in the “brakes and clutch” mode (Figure 9). In the “brakes and clutch” mode, in order to ride as slowly as possible, participants had fewer cumulated deviations at 6 a.m. (124289.75±67988.35°) and 10 a.m. (128391.24±64652.83°) than at 2 p.m. (148129.24±74593.61°) and 6 p.m. (162242.37±83843.09°), which were caused by more important movements of the handlebars in the afternoon.

#### Emergency braking

Before any analysis of motorcycling performance in the emergency braking test, it was necessary to check whether the participants could perform the exercise at the correct speed. Participants respected the speed condition imposed by the test, riding at 20.04±1.05 kph and 39.16±3.65 kph at the beginning of the test (F_(1,11)_ = 709.59; *ε* = 1.00; *p*<0.001; partial *η^2^* = 0.98). More interestingly, a significant interaction effect between “speed condition”, “sleep condition” and “time of day” was observed on the initial speed (F_(3,33)_ = 2.92; *ε* = 1.00; *p*<0.05; partial *η^2^* = 0.21). At 40 kph, TSD had a significant effect on the participants’ ability to start the exercise at the correct speed. Participants rode slower at 6 p.m. following a night of TSD (36.60±3.16 kph) than in all the other test sessions. Participants also rode slower at 2 p.m. following a night of TSD (38.43±4.92 kph) than at the same time of day after a normal night’s sleep.

As for the time necessary to stop the motorcycle, a significant effect of “speed condition” was observed (F_(1,11)_ = 211.14; *ε* = 1.00; *p*<0.001; partial *η^2^* = 0.95). Participants stopped faster when riding at 20 kph (1.88±0.59 s) than when riding at 40 kph (3.51±0.83 s).

**Figure 10:**
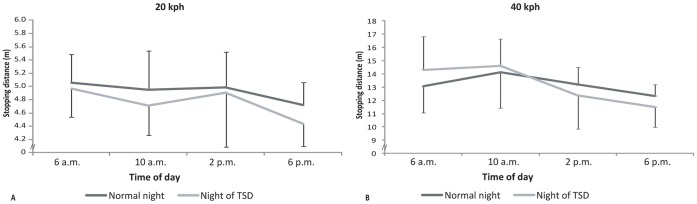
Average stopping distance (m) of the participants measured at 20 kph (A) and at 40 kph (B) at 6 a.m., 10 a.m., 2 p.m., and 6 p.m. after either a normal night’s sleep (Normal night) or a night of TSD (Night of TSD).

As for the distance necessary to stop the motorcycle, a significant effect of “time of day” was observed (F_(3,33)_ = 9.75; *ε* = 0.88; *p*<0.001; partial *η^2^* = 0.47). Participants stopped in a shorter distance at 6 p.m. (8.00±4.06 m) than at all the other times of the day. Less distance was also necessary to stop at 2 p.m. (8.62±4.48 m) than at 6 a.m. (9.09±4.91 m) and 10 a.m. (9.35±5.34 m). The stopping distances also depended on the “speed condition” (F_(1,11)_ = 548.39; *ε* = 1.00; *p*<0.001; partial *η^2^* = 0.98). Participants stopped in a shorter distance at 20 kph (4.34±0.53 m) than at 40 kph (13.19±2.21 m). A significant interaction effect between “speed condition” and “time of day” (F_(3,33)_ = 7.51; *ε* = 1.00; *p*<0.001; partial *η^2^* = 0.41) was observed on the stopping distances. The stopping distances were always shorter when riding at 20 kph than when riding at 40 kph (Figure 10). When the tests were carried out at 40 kph, participants stopped in a shorter distance at 2 p.m. (12.80±2.01 m) and 6 p.m. (11.91±1.27 m) than at 6 a.m. (13.67±2.32 m) and 10 a.m. (14.37±2.36 m).

To have further information, it would be of interest to determine whether the effects observed on the stopping distances were induced by changes in reactions or changes in the braking part of the manoeuvre.

On the one hand, the distances measured during the reaction part of the manoeuvre were influenced by “time of day” (F_(3,33)_ = 3.83; *ε* = 0.88; *p*<0.05; partial *η^2^* = 0.26). Participants needed less distance before pressing the brakes at 6 p.m. (2.64±0.97 m) than at 6 a.m. (2.99±1.18 m), at 10 a.m. (3.01±1.45 m) or at 2 p.m. (2.92±1.24 m). In addition, “speed condition” had a significant effect on the reaction distances (F_(1,11)_ = 356.55; *ε*1.00; *p*<0.001; partial *η^2^* = 0.97). Participants needed less distance before pressing the brakes when riding at 20 kph (1.85±0.40 m) than when riding at 40 kph (3.93±0.80 m). A significant interaction effect between “sleep condition” and “speed condition” (F_(1,11)_ = 12.04; *ε* = 1.00; *p*<0.01; partial *η^2^* = 0.52) was observed. During the tests carried out at 20 kph, participants needed more distance before pressing the brakes following a normal night’s sleep (1.98±0.43 m) than after a night of TSD (1.71±0.31 m).

On the other hand, the statistical analysis indicated that the braking distances were influenced by “time of day” (F_(3,33)_ = 5.43; *ε* = 0.84; *p*<0.01; partial *η^2^* = 0.33). The braking distances were longer at 6 a.m. (5.99±3.83 m) than at 6 p.m. (5.43±3.20 m) and also longer at 10 a.m. (6.07±3.88 m) than at 2 p.m. (5.65±3.42 m) and at 6 p.m. In addition, the braking distances were influenced by “speed condition” (F_(1,11)_ = 432.48; *ε* = 1.00; *p*<0.001; partial *η^2^* = 0.97). Participants had longer braking distances during the tests carried at 40 kph (9.12±1.77 m) than during those at 20 kph (2.45±0.27 m). An interaction effect between “time of day” and “speed condition” on the braking distances was observed (F_(3,33)_ = 3.73; *ε* = 0.84; *p*<0.05; partial *η^2^* = 0.25). At 40 kph, the braking distances were longer at 6 a.m. (9.46±2.19 m) and at 10 a.m. (9.70±1.78 m) than at 2 p.m. (8.86±1.56 m) and at 6 p.m. (8.47±1.25 m).

#### Crash avoidance

As previously, the target speed of 20 or 40 kph requested at the beginning of the test was not reached exactly (F_(1,11)_ = 1225.72; *ε* = 1.00; *p*<0.001; partial *η^2^* = 0.99). Similar tendencies occurred for the crash avoidance test as participants rode at 19.52±1.27 kph and 37.30±3.36 kph. The statistical analysis also indicated an interaction effect between “sleep condition”, “instruction given”, and “speed condition” (F_(1,11)_ = 5.91; *ε* = 1.00; *p*<0.05; partial *η^2^* = 0.35) on the speed recorded at the beginning of the manoeuvre. Following a normal night’s sleep, when the manoeuvres were performed at 40 kph on the opposite side from the LED, recorded speeds (37.33±2.44 kph) were higher than in the manoeuvres performed at the same speed but on the same side as the LED following the night of TSD (36.96±4.29 kph). Moreover, following the night of TSD, when the manoeuvres were performed at 40 kph on the opposite side from the LED, the recorded speeds (37.66±3.54 kph) were higher than in the manoeuvres performed at 40 kph on the same side after a normal night’s sleep (37.27±2.88 kph) and also after the night of TSD.

As for the time needed to perform the crash avoidance manoeuvre, a significant effect of “speed condition” was observed (F_(1,11)_ = 97.52; *ε* = 1.00; *p*<0.001; partial *η^2^* = 0.89). Participants took more time to perform the manoeuvre when riding at 20 kph (2.48±0.54 s) than when riding at 40 kph (2.01±0.38 s). More interestingly, an interaction effect between “sleep condition” and “time of day” was observed on the time needed to perform the crash avoidance manoeuvre (F_(3,33)_ = 2.97; *ε* = 1.00; *p*<0.05; partial *η^2^* = 0.21). Participants performed the crash avoidance manoeuvre more slowly at 2 p.m. following the night of TSD (2.38±0.58 s) than during all the test sessions performed after a normal night’s sleep (2.27±0.62 s at 6 a.m.; 2.20±0.49 s at 10 a.m.; 2.25±0.46 s at 2 p.m.; 2.17±0.49 s at 6 p.m.) and the other test sessions following the night of TSD (2.19±0.47 s at 6 a.m.; 2.22±0.56 s at 10 a.m.; 2.25±0.47 s at 6 p.m.). Following a normal night’s sleep, participants were also significantly faster at performing this manoeuvre at 6 p.m. than at 6 a.m.

To have further information, it would be of interest to determine whether the effects observed on the avoidance times were induced by changes in reactions or changes in the swerving part of the manoeuvre.

On the one hand, the reaction times were influenced by a significant effect of “time of day” (F_(3,33)_ = 10.99; *ε* = 1.00; *p*<0.001; partial *η^2^* = 0.50). Participants’ reaction times were slower at 6 a.m. (0.41±0.15 s) than at 10 a.m. (0.36±0.11 s), 2 p.m. (0.36±0.10 s), and 6 p.m. (0.36±0.11 s). The reaction times measured during the crash avoidance tests were influenced by “instruction given” (F_(1,11)_ = 11.11; *ε* = 1.00; *p*<0.01; partial *η^2^* = 0.50). Participants initiated their manoeuvres more quickly during the tests performed on the same side as the LED (0.36±0.11 s) than during the tests on the opposite side from the LED (0.38±0.12 s). The “speed condition” also influenced reaction times (F_(1,11)_ = 6.57; *ε* = 1.00; *p*<0.05; partial *η^2^* = 0.37). Participants reacted more slowly when the manoeuvres were performed at 20 kph (0.39±0.11 s) than when they were performed at 40 kph (0.36±0.12 s). Interestingly, the statistical analysis revealed an interaction effect between “instruction given” and “direction of the manoeuvre” (F_(1,11)_ = 5.61; *ε* = 1.00; *p*<0.05; partial *η^2^* = 0.34). Participants reacted more quickly during the manoeuvres performed on the same side as the LED when that was the left side (0.35±0.10 s) than during the manoeuvres performed under the same conditions when the side was the right side (0.38±0.12 s), and during those performed on the opposite side from the LED when the LED was on the left side (0.39±0.13 s) than when it was on the right (0.38±0.12 s).

On the other hand, the swerving times were influenced by a significant effect of “speed condition” (F_(1,11)_ = 92.42; *ε* = 1.00; *p*<0.001; partial *η^2^* = 0.89). Participants took more time to adjust their trajectory when riding at 20 kph (2.11±0.59 s) than during the tests performed at 40 kph (1.65±0.42 s).

As for the distances necessary to perform the crash avoidance manoeuvre, a significant effect of “speed condition” was observed (F _(1,11)_ = 159.98; *ε* = 1.00; *p*<0.001; partial *η^2^* = 0.94). Participants needed less distance to perform the manoeuvre when riding at 20 kph (13.83±3.06 m) than when riding at 40 kph (20.29±3.41 m).

## Discussion

The aim of this study was to investigate whether PTW handling capabilities – measured by means of the efficiency of emergency manoeuvres – depended on prior sleep deprivation and time of day. To our knowledge, this study is the first that has been performed in real world conditions on an instrumented motorcycle to evaluate the effects of these disturbance factors. Our major results indicate that motorcycle control at low speed depends on the time of day, with a marked improvement in performance throughout the day. Emergency braking performance is affected at both speeds by time of day, with poorer performance (longer total stopping distances, reaction times and braking distances) in the morning, and also by TSD, when measurements were obtained at 40 kph (incorrect initial speed). Except for a tendency to deviate from the initial speed observed after the night of TSD, it seems that crash avoidance capabilities were unaffected by either time of day or TSD.

Body temperature can be considered as the gold standard of individual rhythmicity. In this study, temperature values were recorded in order to check the body’s circadian evolution. As classically reported, oral temperature evolved according to a circadian rhythm following a normal night’s sleep [37]. This attests to the fact that the participants selected for this study presented a clear circadian rhythmicity with minimal values observed in the early morning and a continuous rise during the day to reach maximal values in the late afternoon. The diurnal fluctuation of body temperature was preserved on the day following the night of TSD, but with lower amplitude due to a decrease in the values recorded at 6 p.m. This observation is in agreement with previous studies which reported an amplitude decrease in body temperature after sleep deprivation, mainly due to a reduction in the acrophase of the rhythm, after 36 hours of wakefulness [47].

The participants’ sleepiness level decreased throughout the day after a normal night’s sleep. This temporal evolution of sleepiness is in agreement with the well-described rhythm of vigilance [48]. After a night of TSD, participants felt sleepier than after a normal night’s sleep. However, their sleepiness level then remained stable throughout the day. This confirms that lack of sleep increases sleepiness through the accumulation from the homeostatic process mentioned in different models [49]. More precisely, the high scores observed in the Epworth Sleepiness Scale in the morning confirmed the bathyphase of the circadian rhythm of vigilance [50]. In the afternoon, the combination of the increased time for which participants had been awake and the repetition of the test sessions may have contributed to the higher level of sleepiness.

Motorcycling handling capabilities were evaluated in three different types of tests. The stability test at low speed was performed in two different modes. In the “slow motion” mode, the speed was fixed. As a consequence, the only way for the participants to improve their handling capabilities in this mode was related to their actions on the handlebars. In this control mode, time of day and sleep deprivation had no effect on the participants’ performance. This task was quite easy for the participants, which may explain why it was not sensitive to the effects of these disturbance factors. In the “brakes and clutch” mode, participants had to reduce their speed by pressing the brake lever and pedal. In passing we should note that their performance was dependent not only on the speed attained but also on the deviation from the course. The average speed constantly decreased throughout the day, which means that the participants were better in the afternoon than in the morning. However, due to the dynamic characteristics of a PTW [51], the decrease observed in speed in the afternoon was accompanied by an increase in deviations [52]. In comparable situations to this test, PTW riders have to take care when going out of their garages or when parking their PTWs in the early morning. They have less control of the PTW at low speed, so they risk hitting obstacles. From a general point of view, PTW riders fail to follow a precise course in the morning. Consequently, particular caution must be adopted in dangerous situations such as riding between rows of cars. Sleep deprivation had no effect on stability at low speed in either the “slow motion” or the “brakes and clutch” mode in our study. The crash risk was a limit for that test, as below a certain speed the participants could not keep the motorcycle upright [53]. Moreover, as this test was particularly motivating and challenging, it is possible that participants’ investment was more important, which could have limited the impact of sleep deprivation [54]–[56].

The results obtained in the emergency braking test confirmed that stopping distance increases as speed increases [57]–[58]. At 40 kph, the participants stopped 9 m (+203%) further on than at 20 kph. In areas with a lot of uncertainty such as city centres, PTW riders should respect speed limits in order to reduce the risk of crashing, as even at the current speeds in such areas they need between 4 and 15 m to stop their vehicle. These stopping distances, corresponding to average braking decelerations of approximately 4.41 m/s^2^ at 40 kph, are in agreement with measurements in PTWs equipped with ABS [3], [59]–[60]. Nevertheless, the average braking decelerations observed in our study were quite low in comparison to the values observed in these previous studies (from 6.19 m/s^2^ to 8.53 m/s^2^). The fact that the initial velocity reached by the riders to achieve the manoeuvre differs between our study (40 kph) and previous work (60 kph –100 kph) partly explains the low values of average braking deceleration observed. The participants were asked to avoid a high level of risk, but nevertheless the ABS/CBS system was sometimes triggered, which proves that they tried to achieve good results in the tests. In our study, the analysis of the emergency braking task allowed us to distinguish between the reaction and the braking tasks. Consequently, the contribution of the mechanisms related to signal detection and those related to execution of the global manoeuvre can be determined. The stopping distance decreased during the day at both speeds. This performance improvement, observed at 20 kph (−0.35 m between 6 a.m. and 6 p.m.) and at 40 kph (−1.60 m between 6 a.m. and 6 p.m.) was determined by both (i) an improvement in reaction time and (ii) greater efficiency in braking. These results can be connected to those generally obtained in laboratories, which report an improvement in reaction time and motor efficiency throughout the day [60]–[65]. The average braking reaction time measured in our study (0.37 s) is in agreement with those previously observed in PTW riders [3], [60], [66]–[68]. Nevertheless, our results highlight that, as in laboratory conditions [64], PTW riders have to be aware that their reactive capacities are slower during morning rides than in the afternoon, leading to longer stopping distances. Even if the performance impairment observed in the early morning in our study remained limited, these results require consideration when interpreting the outcomes, as the PTW riders were highly trained on this task and it was, in addition, realised at low speed. The increase in reaction time observed at 6 a.m. (±0.05 s) corresponded to a small increase in the total stopping distance (±1.60 m at 40 kph). However, this aspect is particularly important, as it is well known that in real world situations, as well as there being concurrent distractions and without the motivation to perform for an experiment, adherence to the road is lower in the morning due to a higher humidity rate, and this can also impair braking performance [58], [60]. In the present study, no effect of sleep deprivation was observed on braking performance. Our results can be compared with those obtained in a previous study indicating longer brake reaction times after insufficient sleep in a laboratory test but not in a road tracking test [69]. Moreover, if the effects of alcohol consumption and TSD can be compared [70]–[71], it must be added that in a comparable task (emergency braking at 12–18 mph), other authors also did not observe any significant deleterious effect of elevated blood alcohol concentration (0.08 g.dl^−1^) on response times and total stopping distances [72]. It seems that, for this exercise as well, the risk of a fall was the limitation, and that the use of a PTW equipped with an ABS/CBS system made it possible for the participants to maintain their performance at an adequate level despite lack of sleep. It can be added that even if sleep loss makes it difficult to sustain alertness, attention or psychomotor vigilance [73], established executive tasks partly remain unaffected [74]. It is likely that the magnitude of performance impairment is directly related to the amount of prolonged and sustained attention that a particular cognitive task requires [75]. Consequently, as suggested previously, greater impairment may be expected from less experienced riders, on less familiar roads, and/or with more complex and novel tasks [72].

In the crash avoidance task, the participants needed an average distance of 13.83±3.06 m and 20.29±4.31 m to perform the manoeuvre at 20 kph and 40 kph, respectively. These results are in agreement with previous studies and confirm that, up to 40 kph, and without any other concurrent distraction, it takes a shorter distance to achieve an emergency stop than to carry out a swerving manoeuvre to avoid a crash [76]. Moreover, regardless of the speed condition, recordings of avoidance times and distances indicate that participants were more efficient in the afternoon than in the early morning after a normal night’s sleep. The manoeuvres were achieved more quickly at 6 p.m. than at 6 a.m., even if the avoidance distances did not change throughout the day. This improvement observed in the avoidance times is mainly caused by an improvement in reaction times. Participants needed between 0.41±0.15 s at 6 a.m. and 0.36±0.11 s at 6 p.m. to initiate their manoeuvre after detecting the LED flash. These data confirm those obtained in laboratory situations, reporting that in a two-choice reaction time test, the delay observed between a visual signal and any motor response is between 0.30 s and 0.40 s [77]. The distance travelled at 40 kph during the mean reaction time (3.74±1.33 m) for the avoidance task was also comparable with the results of a previous study [68]. Reaction times measured during manoeuvres performed on the same side as the LED were shorter than those recorded on the opposite side (+6%). These data confirm that the more complex the task, the longer the reaction time [78]. In addition, participants reacted faster during manoeuvres performed on the same side as the LED when this was the left side than during all the other avoidance manoeuvres. This observation confirms the idea of a preferential avoidance side such as has been proposed previously [68]. The fact that riders use the right-hand side of the road in France and regularly have to perform more avoidance manoeuvres on the left than on the right (the door of a parked car could open, a vehicle could suddenly emerge or there could be a late lane change), could contribute towards explaining our results. TSD had very limited effects on the different parts of the crash avoidance manoeuvre. This absence of perturbation can partly be explained by the test settings. The tests used in this study were very short (15–20 s) and for the purposes of the experiment were carried out without any distraction, which is not the case when motorcycling in the real world for longer distances. Moreover, we should add that even if there were some uncertainty related to the side on which the manoeuvre had to be performed, the participants knew that they had to do something when entering the exercise zone. As a consequence, they were already in an alert state, and were waiting for a signal. Therefore the practical significance of these results must be interpreted in the context of the contrived experimental conditions. Despite the well-known deleterious effects of circadian rhythmicity and sleep deprivation, the participants may have been able to maintain the initial level of the test results. The swerving manoeuvre was the most complex exercise in this study. As previously mentioned, it required riders to maintain speed and control of the motorcycle while approaching the hazard, to respond immediately to the light signal and then to make the correct manoeuvre decision based on their perception and interpretation of the signal [72]. It has already been suggested that compensatory mechanisms between the various performance components may be set up in such complex tasks to limit the deleterious effects of disturbance factors (time of day or sleep deprivation) [79]. Moreover, we noted that even if their objective performance was not affected by the lack of sleep, the participants modified their riding strategy and failed to respect the imposed speed in order to complete the exercise.

In conclusion, this study was the first aimed at evaluating the influence of time of day and sleep deprivation on PTW riders’ handling capabilities using an instrumented motorcycle. Our results demonstrate that performances recorded while handling at low speed and carrying out emergency braking tasks were significantly impaired, while performances remained stable in the crash avoidance task. In everyday conditions, PTW riders have to be aware that their handling capabilities are limited in the early morning and/or after sleep deprivation, which can increase the risk of falls and of being involved in a road accident. Furthermore, despite the high sleepiness level recorded after the night of TSD, the participants could maintain their initial performance level for short duration tasks which included a high risk of falling. However, whereas the tests proposed in this study consisted of short duration tasks, riders take part in monotonous and prolonged rides in real world conditions. As has already been shown for car driving, the influences of time of day and sleep deprivation should be pronounced in such situations. To analyse these effects further, the development of PTW riding simulators, allowing longer test sessions under various environmental conditions without any risk of falling, is now of particular interest.
